# Caesarean deliveries and double burden of malnutrition: a multicountry analysis in South and Southeast Asia

**DOI:** 10.1093/pubmed/fdaf117

**Published:** 2025-09-19

**Authors:** Ashis Talukder, Matthew Kelly, Darren Gray, Haribondhu Sarma

**Affiliations:** National Centre for Epidemiology and Population Health, Australian National University, Canberra, ACT 2600, Australia; Statistics Discipline, Science Engineering and Technology School, Khulna University, Khulna 9208, Bangladesh; National Centre for Epidemiology and Population Health, Australian National University, Canberra, ACT 2600, Australia; Population Health Program, QIMR Berghofer Medical Research Institute, Brisbane, Queensland 4006, Australia; National Centre for Epidemiology and Population Health, Australian National University, Canberra, ACT 2600, Australia

**Keywords:** breastfeeding practices, caesarean section (C-section), double burden of malnutrition (DBM), maternal obesity, multilevel logistic regression, south and Southeast Asia

## Abstract

**Background:**

The increasing prevalence of caesarian section (C-section) births in South and Southeast Asia poses potential public health challenges by influencing maternal and child nutrition. These changes may contribute to the growing double burden of malnutrition (DBM), where maternal overweight/obesity coexists with child undernutrition. This study explores how C-section deliveries are linked to household-level DBM in three countries in this region. Understanding this link is key for developing effective interventions to improve maternal and child nutrition and reduce health burdens.

**Methods:**

We analysed 2022 Demographic and Health Survey (DHS) data from Bangladesh, Cambodia, and Nepal, including women aged 15–49 with at least one child, with available nutritional and delivery mode data. Chi-square tests, analysis of variance, and two-level logistic regression were used to assess the association between C-sections and DBM.

**Results:**

C-section deliveries were linked to a significantly higher risk of DBM in Bangladesh and Nepal. Delayed breastfeeding initiation after C-section further increased this risk. Urban households showed higher DBM rates, while longer breastfeeding duration was protective.

**Conclusion:**

To reduce DBM, policies should focus on limiting unnecessary C-sections, promoting early and sustained breastfeeding, and supporting maternal postpartum health—especially in urban areas where risks are higher. Understanding local factors is crucial for effective interventions.

## Introduction

The health and nutritional impact of caesarean section (C-section) births on mothers and children is a major concern in maternal and child health. While C-sections are often necessary to prevent maternal and neonatal morbidity and mortality, they can also lead to significant health and nutritional challenges for both mothers and children.^1^^,^^2^ C-section deliveries may increase the risk of maternal complications, including infections, prolonged recovery times, and difficulties in subsequent pregnancies.^3–6^ These complications can interfere with optimal feeding practices, particularly breastfeeding, which is essential for child nutrition and immune protection.^7^^,^^8^ Additionally, the surgical nature of C-sections may disrupt early mother–child bonding, potentially affecting the successful initiation of breastfeeding.^8–10^ This can contribute to inadequate child nutrition and an increased risk of malnutrition in early childhood.^11^^,^^12^

Infants delivered via C-section are less likely to be exclusively breastfed, often due to maternal recovery challenges such as pain, fatigue, and delayed lactation initiation.^13–15^ This can lead to increased reliance on formula feeding,^14–17^ which may not provide the same immune and nutritional benefits as breast milk, potentially affecting child growth and metabolism and increasing the risk of undernutrition.^18–21^ Additionally, disruptions in gut microbiota composition among C-section-delivered infants can impair nutrient absorption and increase the risk of obesity, metabolic syndrome, and other noncommunicable diseases later in life.^22–24^

The postpartum period after a C-section presents unique challenges for mothers, including longer recovery times, limited mobility, and metabolic changes such as insulin resistance and altered lipid metabolism, which increase the risk of excessive weight gain and obesity.^12^^,^^25–28^ Postpartum fatigue and psychological stress may also influence dietary habits, worsening weight retention.^28^ Recent evidence shows that mothers who undergo C-section deliveries are more likely to experience postpartum overweight and obesity.^29–31^ For example, one study found that 21.5% of C-section mothers were obese compared to 13.2% of those with vaginal births, and their children also had a higher risk of obesity by age three.^32^ A meta-analysis of 28 studies further reported a 34% increased risk of childhood obesity among caesarean-delivered children.^33^ In low-income settings such as Ghana, C-section births have also been linked to lower height-for-age *z*-scores, indicating a greater risk of stunting.^18^ Similarly, India’s National Family Health Survey (NFHS)-5 (2019–21) data shows that children born via C-section experience higher rates of stunting, wasting, and underweight than those born vaginally.^34^ As maternal overweight is also a known risk factor for C-section delivery,^35–37^ these findings suggest a plausible bidirectional pathway linking C-section births to the double burden of malnutrition (DBM) at the household level—where overnutrition in mothers and undernutrition in children coexist.

Although a few studies have highlighted this potential link, the evidence remains limited. One previous study examined the association between C-section births and household-level DBM using an earlier wave of Bangladesh Demographic and Health Survey (DHS) data,^12^ but it was restricted to a single-country context and did not consider broader regional patterns. Moreover, household nutrition dynamics may have shifted in recent years due to changing health systems, rising urbanization, and evolving food environments, making updated evidence essential. Our study responds to this need by analysing the most recent DHS data from Bangladesh, Cambodia, and Nepal to assess the relationship between C-section deliveries and household-level DBM. By incorporating geographic and urban–rural dimensions, this study offers a regional and comparative perspective, providing timely evidence to inform integrated maternal and child nutrition strategies across South and Southeast Asia.

## Methods

### Data source and study population

We analysed data from the most recent DHSs conducted in 2022 in Bangladesh, Cambodia, and Nepal. These countries were purposefully selected based on three key criteria. First, all three are undergoing rapid nutritional transitions, characterized by a shift from traditional diets to increased consumption of processed and unhealthy foods—contributing to DBM at the household level.^38–40^ Second, they represent a range of socioeconomic and public health contexts, providing an opportunity to examine how differing stages of development influence the relationship between caesarean births and household nutritional outcomes.^41^ Third, the DHS datasets from these countries offer rich, standardized, and nationally representative data on maternal and child health and nutrition, including anthropometric and health indicators essential for our analysis, and are collected using rigorous, comparable methodologies supported by the MEASURE DHS project.^42^

The DHS employs a robust multistage cluster sampling design, ensuring every unit has a defined probability of selection. Clusters, often enumeration areas (EAs) from recent population and housing censuses, were selected proportionally. Households within these clusters were then systematically sampled. While sampling designs vary slightly by country, most surveys stratify samples by urban and rural areas and geographical or administrative regions.

The height and weight of both mothers and children were measured by trained personnel following standardized procedures used in the DHS. Digital weighing scales (e.g. SECA, a well-known brand for producing digital weighing scales or UNICEF models) were used to measure weight, while height and length were measured using portable measuring boards (such as ShorrBoard® or equivalent UNICEF devices). Children under 24 months were measured lying down (recumbent length), and older children and adults were measured standing. To ensure measurement accuracy, a subset of children were randomly selected for repeat measurements, and extreme anthropometric values were rechecked according to DHS guidelines.^43–45^

For this study, we selected mother–child pairs from the DHS survey, focusing on nonpregnant women aged 15–49 years who had at least one child. We included those pairs with valid measures of weight and height for both the mother and her child. For assessing the association between C-section delivery and DBM, we included pairs where the mother had completed nutritional information and where data were available on the mode of delivery—specifically, whether the child was delivered via C-section or vaginal birth (normal delivery). Additionally, we ensured that nutritional information for the corresponding child was accessible. If a mother had more than one child, we selected the most recent birth with complete nutritional and delivery data. Observations with missing or flagged data for either maternal BMI or child nutritional status were excluded to ensure data quality and to accurately calculate the DBM outcome. For instance, in Bangladesh, 4652 mother–child pairs were initially eligible, but 520 pairs (11.2%) were excluded due to missing or flagged values, resulting in a final analytic sample of 4132 pairs. Similarly, after applying the same eligibility criteria, the final analytic samples were 3836 in Cambodia and 2596 in Nepal.

### Outcome variable

Our primary outcome was the household-level DBM, defined as a pair where the mother was overweight or obese (BMI ≥ 23.0 kg/m^2^ for Asians)^46^ and the child was undernourished (stunted, wasted, or underweight, with *z*-scores < −2). We focused on this pattern because it is more prevalent in these settings and widely documented in the literature.^34^ The reverse pattern—undernourished mother and overnourished child—was rare in our data and thus excluded from analysis.

### Exposures and covariates

The main exposure variable was whether the child was delivered via C-section. We classified this as ‘Yes’ for C-section births and ‘No’ for vaginal deliveries. Based on existing literature, we included several covariates. At the individual level, we considered maternal age (15–23, 24–30, and >30 years), early initiation of breastfeeding (within or after 1 hour), breastfeeding duration (in months), and birth order (first birth, second birth, otherwise). At the cluster level, we included place of residence (urban/rural) and community-level antenatal care (ANC) coverage. To estimate community ANC coverage, we calculated the proportion of women in each cluster who attended at least four ANC visits during pregnancy and categorized them as either low (<50%) or high (≥50%).

### Statistical analysis

We applied DHS sampling weights to account for the complex survey design, including stratification and clustering. Descriptive statistics and bivariate analyses [chi-square and analysis of variance (ANOVA)] were conducted. To address clustering, we used two-level multilevel logistic regression models with the ‘glmer’ function in R, including interaction terms between C-section and delayed breastfeeding initiation, and between C-section and household wealth. Adjusted odds ratios, standard errors, and 95% CIs for interactions were obtained using the delta method via the ‘margins’ package. We checked for multicollinearity (Variance Inflation Factor (VIF) < 5) and assessed model fit using the Hosmer–Lemeshow test. We also used the Getis-Ord Gi* statistic to identify spatial clusters of DBM and C-section deliveries at the district level.^46–48^ This method allowed us to detect statistically significant hot spots by comparing local and global means.^46^ More details on the statistical analysis are available in the online supplementary material.

## Results

**Figure 1:**
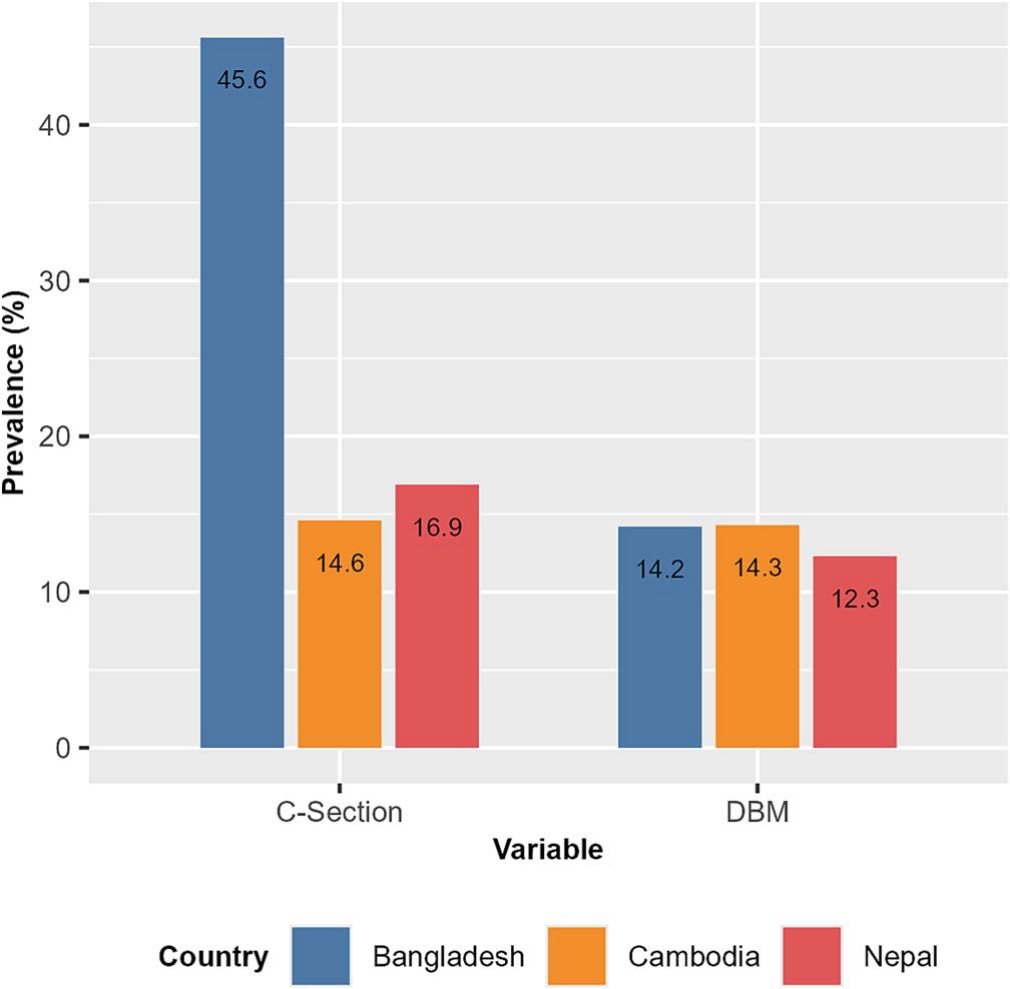
Prevalence of DBM (response variable) and C-section (exposure variable) for Bangladesh, Cambodia, and Nepal.

The prevalence of DBM and C-section deliveries revealed distinct patterns across the three countries (Fig. 1). Bangladesh had an alarmingly high prevalence of C-section deliveries (45.6%), indicating a possible overuse of surgical births that far exceeded the WHO recommended threshold. In contrast, Cambodia (14.6%) and Nepal (16.9%) had much lower rates, suggesting relatively more conservative use of C-sections. Despite these differences, DBM prevalence remained somewhat similar between Bangladesh (14.2%) and Cambodia (14.3%), with Nepal slightly lower (12.3%).

Table 1 highlights the prevalence of DBM across the selected countries. DBM was prevalent among mothers who had C-section deliveries—15.4% in Bangladesh, 16.4% in Cambodia, and 25.2% in Nepal—indicating that surgical birth may disrupt early maternal and child nutritional dynamics. The increasing prevalence with maternal age, especially among those aged 30 or above, may reflect cumulative nutritional risks and lifestyle changes. Households with delayed initiation of breastfeeding (after 1 hour) and those in urban areas had higher DBM prevalence, suggesting that surgical delivery practices and urban dietary transitions could be contributing factors. Lower community ANC coverage had higher DBM prevalence in Nepal and Cambodia, possibly pointing to gaps in prenatal nutrition education and follow-up. Moreover, households affected by DBM had shorter mean breastfeeding durations—14.5 months in Bangladesh, 13.7 months in Cambodia, and 16.2 months in Nepal—supporting the hypothesis that reduced breastfeeding may limit child nutrition while maternal overweight persists. These findings suggest that both clinical practices and broader socioenvironmental contexts shape the dual burden of malnutrition within households.

**Table 1:** Prevalence of double burden of malnutrition (DBM) at household level by background characteristics in Bangladesh, Cambodia, and Nepal along with associated *P*-values obtained from chi-square test (categorical variable) and ANOVA (continuous variable).

Variable	Type	Bangladesh		Cambodia		Nepal	
		DBM *n* (%)	*P*-value	DBM *n* (%)	P-value	DBM *n* (%)	*P*-value
**C-section (CS)**	**Categorical**		<.05				<.01
No		167 (12.2)		262 (13.4)	.144	258 (11.1)	
Yes		177 (15.4)		56 (16.4)		170 (25.2)	
**Mother’s age (year)**	**Categorical**		<.05		<.05		
15–23		170 (12.5)		89 (13.4)		101 (11.8)	.382
24–30		242 (14.4)		203 (13.6)		153 (12.5)	
30+		177 (16.2)		270 (16.1)		74 (14.4)	
**Early initiation breastfeeding (EIB)**	**Categorical**		<.05		<.01		<.01
Within 1 hour		104 (12.1)		273 (13.3)		186 (12.3)	
After 1 hour		246 (15.4)		30 (20.4)		163 (17.2)	
**Birth order**	**Categorical**				.223		.660
First birth		224 (14.1)	.363	184 (14.1)		131 (12.1)	
Second birth		212 (15.2)		189 (13.9)		103 (12.6)	
Otherwise		153 (13.3)		189 (16.1)		94 (13.5)	
**Place of residence**	**Categorical**		<.05		<.01		<.05
Rural		377 (13.4)		300 (12.1)		146 (11.1)	
Urban		212 (16.0)		262 (19.4)		182 (14.2)	
**Community ANC coverage**	**Categorical**		.455		<.10		<.05
Low		375 (14.6)		80 (14.9)		272 (13.3)	
High		214 (13.7)		469 (11.5)		51 (10.1)	
**Months of breastfeeding**	**Continuous**	**Mean (SD):** **14.5 (5.64)**	<.01	**Mean (SD):** **13.7 (5.21)**	<.01	**Mean (SD): 16.2 (6.22)**	<.05

Table 2 presents adjusted odds ratios (AORs) from a two-level logistic regression of factors associated with DBM. In Nepal, children born via C-section were 72% more likely to live in a household experiencing DBM (AOR = 1.72, 95% CI: 1.09–2.70), and in Bangladesh, the likelihood was more than double (AOR = 2.60, 95% CI: 1.55–4.37). This suggests that surgical births may influence early feeding patterns or maternal recovery, with downstream nutritional consequences. Delayed initiation of breastfeeding independently elevated the odds of DBM in all three countries, emphasizing the nutritional importance of early breastfeeding. Shorter breastfeeding duration also showed a negative association with DBM, reinforcing the protective role of breastfeeding. At a contextual level, urban households were more prone to DBM, likely reflecting shifts in diet and lifestyle. The effect of C-sections in urban areas in Nepal and Bangladesh indicate that urbanization and C-section births may together increase DBM vulnerability. Lastly, high community ANC coverage served as a protective factor in Nepal, indicating the potential of community-level interventions in mitigating DBM risks. The Hosmer–Lemeshow test, with insignificant *P*-values, confirms that the multilevel regression models appropriately capture the relationship between DBM and independent variables, supporting the reliability of the findings.

**Table 2:** Multilevel logistic regression model estimates for DBM in Bangladesh, Cambodia, and Nepal.

Variable	Category	Bangladesh		Cambodia		Nepal	
		AOR (95% CI)	*P*-value	AOR (95% CI)	*P*-value	AOR (95% CI)	*P*-value
**Individual factors**							
**C-section (CS)**	**No (ref.)**						
	Yes	2.60 (1.55, 4.37)	<.01	1.71 (0.72, 4.07)	.227	1.72 (1.09, 2.70)	<.05
**Mother’s age (year)**	**15–23 (ref.)**						
	24–30	1.01 (0.72, 1.42)	.934	0.97 (0.63 1.48)	.895	0.96 (0.69, 1.32)	.815
	30+	0.89 (0.58, 1.35)	.576	0.83 (0.50, 1.36)	.483	1.11 (0.73, 1.70)	.621
**Early initiation breastfeeding (EIB)**	**Within 1 hour (ref.)**						
	After 1 hour	1.82 (1.15, 2.88)	<.01	3.12 (1.28, 7.61)	<.05	1.66 (1.26, 2.19)	<.01
**Birth order**	**First birth (ref.)**						
	Second birth	1.16 (0.84, 1.61)	.364	0.86 (0.57, 1.28)	.451	0.97 (0.71, 1.34)	.881
	Otherwise	1.06 (0.71, 1.59)	.779	1.39 (0.88, 2.21)	.158	1.05 (0.72, 1.53)	.798
**Months of breastfeeding**	**Continuous**	0.69 (0.51, 0.94)	<.05	0.47 (0.32, 0.71)	<.01	0.67 (0.51, 0.91)	<.01
**Interaction** $\left(\mathbf{CS}\times \mathbf{EIB}\right)$	$\mathbf{CS}:\mathbf{Yes}\times \mathbf{EIB}:\mathbf{After}\ \mathbf{1}\ \mathbf{hour}$	1.90 (1.07, 3.38)	<.05	1.47 (1.01, 2.14)	<.05	3.26 (1.79, 5.94)	<.01
**Cluster level factors**							
**Place of residence (PR)**	**Rural (ref.)**						
	Urban	2.11 (1.41, 3.16)	<.01	1.80 (1.21, 2.68)	<.01	2.09 (1.47, 2.98)	<.01
**Community ANC coverage**	**Low (ref.)**						
	High	0.96 (0.68, 1.31)	.785	0.97 (0.61, 1.61)	.995	0.58 (0.39, 0.85)	<.01
**Interaction (** $\mathbf{CS}\times \mathbf{PR}\Big)$	$\mathbf{CS}:\mathbf{Yes}\times \mathbf{PR}:\mathbf{Urban}$	1.82 (1.08, 3.06)	<.05	1.52 (0.66, 3.51)	.318	1.92 (1.41, 4.09)	<.01
		$\mathbf{CRE}=\mathbf{0.5}$ **8**		$\mathbf{CRE}=\mathbf{0.69}$		$\mathbf{CRE}=\mathbf{0.63}$	
		$\mathbf{ICC}=\mathbf{0.10}$		$\mathbf{ICC}=\mathbf{0.20}$		$\mathbf{ICC}=\mathbf{0.11}$	
**Hosmer–Lemeshow test**		${\boldsymbol{\mathsf{\boldsymbol{\chi}}}}^{\mathbf{2}}=\mathbf{18.66}$ $\boldsymbol{P}$***-*** $\mathbf{value}=.\mathbf{28}$		${\boldsymbol{\mathsf{\boldsymbol{\chi}}}}^{\mathbf{2}}=\mathbf{11.38}$ $\boldsymbol{P}$**-** $\mathbf{value}=.\mathbf{23}$		${\boldsymbol{\mathsf{\boldsymbol{\chi}}}}^{\mathbf{2}}=\mathbf{5.44}$ $\boldsymbol{P}$**-** $\mathbf{value}=.\mathbf{65}$	

Notes: CRE, cluster random effect. Interaction effects were estimated for C-section (CS) with early initiation of breastfeeding (EIB) and C-section (CS) with place of residence (PR). Estimates were obtained by controlling other covariates in the model.

**Figure 2:**
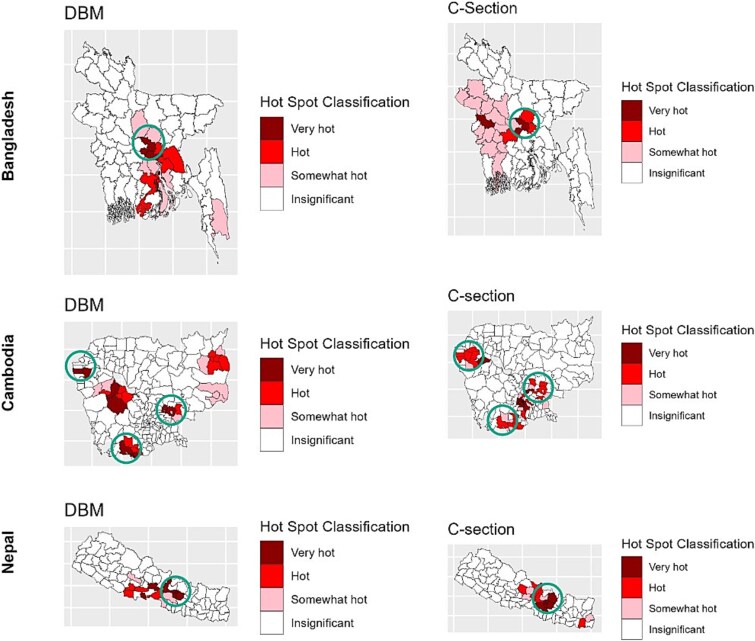
Hot spot analysis for DBM (response variable) and C-section (exposure variable) of Bangladesh, Cambodia, and Nepal. Circles represent common regions for both DBM and C-section. Note: Very hot: hot spot (99% confidence); hot: hot spot (95% confidence); somewhat hot: hot spot (90% confidence).

Figure 2 displays the hot spot analysis results for the prevalence of DBM and C-section deliveries across Bangladesh, Cambodia, and Nepal. The analysis revealed notable geographic overlaps between DBM and C-section hot spots. We identified one region each in Bangladesh and Nepal as a common hot spot where DBM and C-section intersected (Fig. 2). In Cambodia, this overlap was more pronounced, with three regions exhibiting concurrent hot spots. These findings point to a possible spatial relationship between DBM and C-section deliveries in certain areas of the selected countries.

## Discussion

### What is already known on this topic

While some studies suggest that children born via C-section are more likely to live in households experiencing DBM,^12^^,^^49^ a comprehensive regional analysis remains lacking. C-section deliveries can disrupt the infant’s microbiota, increasing their susceptibility to obesity and metabolic disorders later in life,^47^^,^^48^ and potentially contributing to maternal obesity through altered energy metabolism and fat storage.^47^ In resource-limited settings, the higher cost of C-sections can strain household finances, reducing access to nutritious food and healthcare,^50–52^ leading to child undernutrition and prompting reliance on cheaper, energy-dense, low-nutrient foods that promote maternal overweight.^53^^,^^54^ Longer recovery times after C-sections may also affect subsequent pregnancies and overall health,^12^ creating a cycle of poor maternal nutrition. Overweight or obese mothers may, in turn, be more likely to give birth to undernourished children,^55^ perpetuating DBM within households.

### Main findings of this study

C-section deliveries were strongly associated with an increased likelihood of DBM, particularly in Bangladesh and Nepal, where the risk was compounded when breastfeeding was delayed. Urban households across all three countries were more likely to experience DBM, indicating that urbanization can be a contributing factor to the increase in the prevalence of this condition. Furthermore, longer breastfeeding durations appeared to reduce the likelihood of DBM, emphasizing the protective role of extended breastfeeding in mitigating malnutrition. Our analysis also highlighted geographic areas in each country where both high rates of DBM and C-section deliveries were concentrated, suggesting that local factors may be driving these overlapping trends.

### What this study adds

To our knowledge, this is the first region-wide study across Bangladesh, Cambodia, and Nepal to examine the association between C-section delivery and household-level DBM using the most recent nationally representative DHS data. Although the findings may not be fully generalizable to all of South and Southeast Asia due to cultural, dietary, and health system differences, they offer valuable case studies to guide future research in similar contexts. We found that delayed initiation of breastfeeding significantly increased DBM risk across all countries, particularly when combined with C-section deliveries, consistent with previous studies.^12^^,^^49^ One explanation is that C-section births are more likely to delay breastfeeding initiation,^56^^,^^57^ depriving infants of essential early nutrition and immune protection,^58^ while disrupted maternal hormonal and metabolic processes may further raise the obesity risk.^59–61^

Although Bangladesh reports a higher rate of C-section deliveries, the prevalence of DBM remains comparable to that in Cambodia and Nepal. One possible explanation is that C-sections in Bangladesh are predominantly concentrated among urban and wealthier households,^62^^,^^63^ where child undernutrition tends to be lower due to better access to healthcare, improved maternal education, and better infant feeding practices.^64^ While maternal overweight is more common in these groups, the relatively better nutritional status of children may reduce the overall likelihood of DBM. This pattern may help explain why rising C-section rates in Bangladesh have not yet translated into higher DBM prevalence, although continued monitoring is warranted as nutrition transitions progress. We also emphasize that households with a history of C-sections are more likely to experience DBM, which is a key message for guiding public health responses.

We found that extended breastfeeding plays an important role in reducing DBM risk. Households where mothers breastfed longer were significantly less likely to experience DBM, consistent with prior research on benefits for both mothers and children.^65–67^ Longer breastfeeding supplies essential micronutrients for child growth and supports postpartum weight loss in mothers.^68^^,^^69^ Evidence also shows that exclusive breastfeeding for up to 6 months improves child health and nutrition while reducing maternal weight retention.^70^ Promoting optimal breastfeeding is therefore crucial to healthy growth, preventing maternal obesity, and breaking the DBM cycle in vulnerable households.

Urban households were more likely to experience DBM than rural households, consistent with previous studies.^71^ Higher obesity rates among urban women stem from unhealthy diets, sedentary lifestyles, and limited physical activity.^72^^,^^73^ Although urban children generally face lower undernutrition rates, factors such as food insecurity and inadequate services in low-income urban areas still contribute to malnutrition.^74^ We also found that high community-level ANC coverage significantly reduced DBM risk in Nepal but not in Bangladesh or Cambodia, likely reflecting differences in service quality and content. In Nepal, a strong healthcare infrastructure and effective nutrition education through ANC may play a protective role.^75^^,^^76^

Our spatial analysis identified overlapping hot spots of DBM and C-section deliveries, particularly in Cambodia, with distinct clusters in Bangladesh and Nepal as well. These patterns may reflect disparities in healthcare access, socioeconomic conditions, and maternal and child health practices.^77^^,^^78^ Identifying these geographic patterns underscores the need for localized interventions to address both malnutrition and the overuse of C-section deliveries.

This study has several strengths. Using nationally representative DHS data from Bangladesh, Cambodia, and Nepal ensures robust, generalizable findings across diverse settings. Standardized methods and high response rates enhance reliability and comparability. Focusing on countries in rapid nutrition transition provides valuable regional insights into household-level DBM. Multilevel modelling strengthens the analysis by accounting for clustering and contextual factors. Overall, the study addresses a substantial evidence gap on the intersection between C-section deliveries and DBM, offering novel insights with direct implications for maternal and child health programs and policy development in transitioning economies.

### Limitations of this study

This study has some limitations. The cross-sectional design limits causal interpretations. Additionally, we were unable to account for other potentially important factors influencing DBM, such as daily dietary patterns for children under 5 (0–59 months), physical activity, caregiving practices, cultural factors, and detailed pregnancy and birth data, which is not included in the DHS dataset. The absence of confounders like household income and healthcare access may have also influenced the findings. Moreover, using data from only three countries restricts the generalizability of results across South and Southeast Asia, given the diverse socioeconomic and health system contexts. Therefore, caution is advised when applying these findings beyond the countries studied.

## Conclusion

This study identifies a significant association between C-section deliveries and rising prevalence of DBM in Bangladesh and Nepal, highlighting the need to limit unnecessary surgical births through stricter clinical guidelines and health system oversight. Promoting early initiation of breastfeeding after C-sections, alongside postpartum weight management, is a crucial preventive measure. Strengthening community-based ANC services with nutritional counselling can effectively address DBM at the local level. Addressing urban–rural disparities requires improving access to nutritious foods and promoting healthier lifestyles in urban areas. Importantly, policy responses and interventions must be context-specific, accounting for the regional health infrastructure, cultural norms, and geographic differences. Supporting locally driven research and fostering multisectoral collaboration will be key to developing and implementing effective strategies to reduce DBM and unnecessary C-sections in South and Southeast Asia.

## Supplementary Material

Supplementary_file_Updated_fdaf117

## Data Availability

Data is freely available in the public domain with the following link: https://dhsprogram.com/data/dataset_admin/login_main.cfm?CFID=300458265&CFTOKEN=26bd09600ba5696-DE5E82D0-A6C1-5C66-C216C2CD9B9E82D9.

## Notes

**Ashis Talukder**, PhD Researcher

**Matthew Kelly**, Associate Professor

**Darren Gray**, Professor & Program Director of Population Health

**Haribondhu Sarma**, Senior Lecturer/Fellow
