# Supplementary material for: Caesarean deliveries and double burden of malnutrition: a multicountry analysis in South and Southeast Asia
Source: J Public Health (Oxf). 2025 Sep 19;47(4):e546–56. doi: 10.1093/pubmed/fdaf117 (PMC12670005; doi:10.1093/pubmed/fdaf117)
Supplement: Supplementary_file_Updated_fdaf117 [file supplementary_file_updated_fdaf117.pdf]

Supplementary file

Methods

Statistical Analysis

In our analysis, we applied the sampling weights provided in the DHS dataset to account for the complex survey design, which includes stratification and clustering. This approach ensured that the findings were nationally representative and adjusted for design effects, thereby reducing potential biases associated with unequal probabilities of selection. By incorporating these weights, we improved the reliability and generalizability of our estimates, ensuring that they accurately reflected the true distribution of the population across different demographic and socioeconomic groups.

We conducted descriptive analyses to summarise the characteristics of the study population. To explore associations between predictor variables and DBM, we first performed bivariate chi-square tests for categorical variables and analysis of variance (ANOVA) for continuous variables. To account for potential clustering effects, we applied a two-level logistic regression model, defined as:

$$logit(P_{ij}) = \beta_{00} + \beta_{10}x_{ij} + \beta_{01}X_j + V_{0j}$$

Where, $logit(P_{ij}) = \log(\frac{P(Y_{ij}=1)}{1-P(Y_{ij}=1)})$, β_{00} is the fixed effect intercept and V_{0j} is the level-2 residual. The variables x_{ij} and X_j correspond to level-1 (individual-level) and level-2 (cluster-level) variables, respectively, with β_{10} and β_{01} denoting their fixed effects. In this study, covariates were classified as individual-level variables (C-section delivery, mother's Age, initiation breastfeeding, months of breastfeeding and interaction between C-section delivery and initiation of breastfeeding and interaction between C-section delivery and place of residence) and cluster-level variables (place of residence and community ANC coverage). To construct the interaction between C-section delivery and initiation of breastfeeding, we multiplying these two binary variables. This new variable captures whether the effect of C-section delivery on the outcome varies based on whether breastfeeding was initiated timely or delayed. Similarly, to assess the interaction between C-section delivery and place of residence, we generated another interaction term by multiplying these two binary variables. This term allows us to evaluate whether the

association between C-section delivery and the outcome differs between urban and rural settings. Both interaction terms were included in the regression models alongside their main effects and relevant covariates to assess their influence on the outcome variable.

The intra-class correlation coefficient (ICC) was calculated using an empty multilevel model to assess the degree of clustering. A multilevel logistic regression model was deemed appropriate as the ICC exceeded zero. To ensure that the predictors in our models were not highly correlated, we assessed multicollinearity by checking that the variance inflation factor (VIF) was less than 5. We evaluated the fit of the regression model using the Hosmer & Lemeshow test, which compares the predicted probabilities with the observed outcomes across various risk groups. A non-significant result ($p > 0.05$) suggests no significant difference between the observed and expected values, indicating that the model fits the data well. This implies that the model's predictions are reliable and accurately reflect the relationship between the predictor variables and the outcome. All data analyses were conducted using SPSS and R (R Foundation for Statistical Computing, Vienna, Austria) software.

Hot-spot analysis

We used the Getis-Ord (Gi*) statistic to assess spatial autocorrelation and identify clusters of DBM and C-section deliveries at the district level in the selected countries. We applied the local Gi* statistic to quantify the intensity and stability of hotspot clusters. This method compares the local mean rate (i.e., the rates for a specific location and its neighbors) to the global mean rate (i.e., the rates across all locations in the study). The Gi* statistic calculates a Z-score and p-value for each location, which allows us to assess whether the local mean differs significantly from the global mean. Locations with significantly high Z-scores are classified as hotspots, indicating areas where high values are spatially concentrated, and such patterns are unlikely to occur by chance.

Supplementary Fig. 1

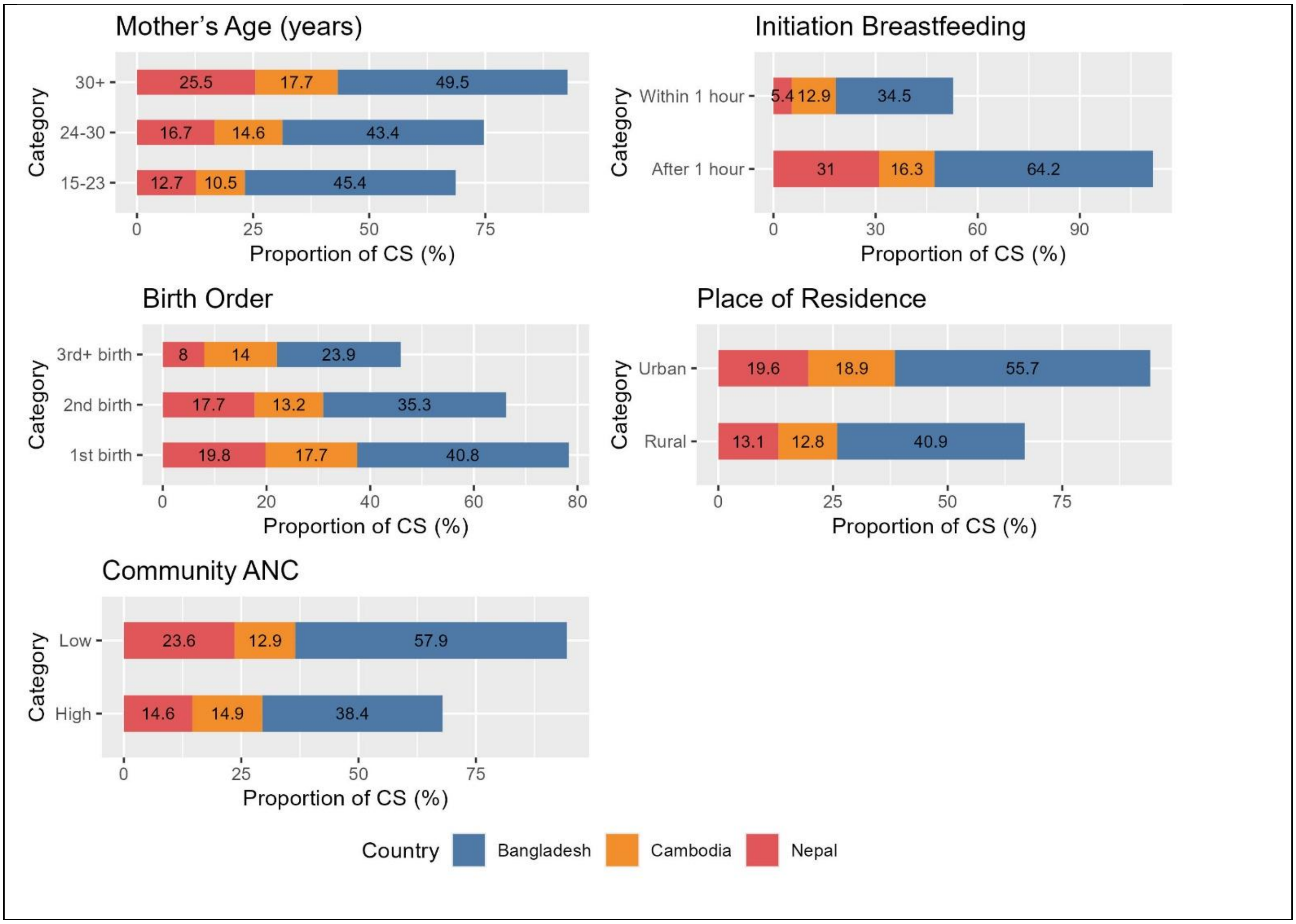

Supplementary Fig. 1: Proportion of C-Section delivery by background characteristics

Fig. 1. Prevalence of DBM (Response variable) and C-section (Exposure variable) for Bangladesh, Cambodia, and Nepal.

Proportion of C-Section Deliveries by Background Characteristics

Supplementary Figure 1 reveals that C-section deliveries are disproportionately concentrated among specific sociodemographic groups across the three countries. The highest proportions were observed among women aged 30 and above—49.5% in Bangladesh, 17.7% in Cambodia, and 25.5% in Nepal—suggesting that older maternal age may be associated with increased obstetric

complications or a preference for medicalised births. First-time mothers consistently showed higher C-section rates, likely reflecting clinical caution during primiparous deliveries. A strong association was evident between delayed breastfeeding initiation and C-sections, particularly in Bangladesh (64.2%) and Nepal (31.0%), possibly reflecting recovery delays that hinder immediate postnatal care. Urban residence was also linked with higher C-section rates, highlighting healthcare system differences or rising demand for surgical procedures in cities. Interestingly, in both Bangladesh and Nepal, women in areas with lower community ANC coverage had higher C-section rates (57.9% in Bangladesh & 23.6% in Nepal), which may reflect either insufficient prenatal risk detection or overuse of surgical delivery in poorly monitored settings. In contrast, Cambodia showed a slightly different pattern, where C-sections were more common in high ANC coverage areas (14.9%), possibly due to better access to surgical services.
